# Whole-Genome Sequence of “*Candidatus* Profftella armatura” from *Diaphorina citri* in Guangdong, China

**DOI:** 10.1128/genomeA.01282-15

**Published:** 2015-11-05

**Authors:** F. Wu, X. Deng, G. Liang, J. Huang, Y. Cen, J. Chen

**Affiliations:** a Laboratory of Insect Ecology/Department of Plant Pathology, South China Agricultural University, Guangzhou, Guangdong, People’s Republic of China; b USDA-ARS, San Joaquin Valley Agricultural Sciences Center, Parlier, California, USA

## Abstract

The genome of “*Candidatus* Profftella armatura” strain YCPA from *Diaphorina citri* in Guangdong, China, was sequenced. The strain has a chromosome of 457,565 bp, 24.3% G+C content, 364 predicted open reading frames (ORFs), and 38 RNAs, and a plasmid, pYCPA54, of 5,458 bp with 23.9% G+C content and 5 ORFs.

## GENOME ANNOUNCEMENT

Asian citrus psyllids (*Diaphorina citri*) transmit the pathogen of citrus Huanglongbing (HLB, yellow shoot disease) which is threatening citrus production worldwide (1, 2). “*Candidatus* Profftella armatura” is a symbiont of *D. citri*. This unculturable beta-proteobacterium inhabits the syncytium of the psyllid bacteriome. A previous study revealed that “*Ca.* Profftella armatura” produced toxins that potentially protected *D. citri* from natural enemies (3). The whole-genome sequence of a Japanese strain of “*Ca.* Profftella armatura” has been reported (3). Here, we report the whole-genome sequence of “*Ca.* Profftella armatura” strain YCPA from *D. citri* in Guangdong Province, China, where HLB was observed over a hundred years ago and is currently endemic.

For sequencing the “*Ca.* Profftella armatura” genome, a colony of *D. citri* was established and maintained in a growth chamber (28 ± 1°C, 60 ± 5% RH, 14:10 h L:D) at South China Agricultural University, Guangzhou, Guangdong, China. DNA was extracted from a single psyllid using a DNeasy blood and tissue kit (Qiagen, Shanghai, China). To meet the quantity requirement for sequencing, whole-genomic DNA was amplified through an Illustra GenomiPhi V2 DNA amplification kit (GE Healthcare, Waukesha, WI, USA) that yielded in 20 µg of DNA. A sample of 10 µg DNA was subjected to sequencing using Illumina MiSeq format (Illumina, San Diego, CA).

A total of 3.98 × 10^7^ reads with a mean of 251 bp per read were generated. *De novo* assembling was performed with CLC Genomics Workbench 7.5. Among the 83,161 large contigs (≥1,000 bp) generated, 2 (456,891 bp and 5,119 bp) were identified to be associated with “*Ca.* Profftella armatura” by standalone BLAST (version 2.2.30) (4) using the genome sequence of “*Ca.* Profftella armatura” strain DC (CP003468.1, CP003469.1) (3) as references. Sequence gaps were filled and genome circularity was proved through the process of designing primers, standard PCR, amplicon sequencing, and sequence assembling. The chromosome of “*Ca.* Profftella armatura” strain YCPA derived from the larger contig with the size of 457,565 bp and G+C content of 24.3%. A plasmid, designated pYCPA54, derived from the smaller contig with the size of 5,458 bp and G+C content of 23.9%. Sequence annotation was performed by the RAST server (http://rast.nmpdr.org/) (5). The chromosome of strain YCPA was predicted to have 364 open reading frames (ORFs) and 38 RNA genes. pYCPA54 was predicted to have 5 ORFs. Both the chromosome of strain YCPA and pYCPA54 shared >99% similarities to the chromosome (CP003468.1) and plasmid (CP003469.1) of the Japanese “*Ca.* Profftella armatura” strain (3).

### Nucleotide sequence accession numbers.

This complete genome sequence has been deposited in DDBJ/EMBL/GenBank under the accession numbers CP012591 (chromosome) and CP012592 (plasmid). The versions described in this manuscript are the first versions, CP012591.1 and CP012592.1.

## References

[1] Bové JM 2006 Huanglongbing: a destructive, newly emerging, centuryold disease of citrus. J Plant Pathol 88:7–37.

[2] Halbert SE, Manjunath KL 2004 Asian citrus psyllids (*Sternorrhyncha*: *Psyllidae*) and greening disease of citrus: a literature review and assessment of risk in Florida. Fla Entomol 87:330–353. doi:10.1653/0015-4040(2004)087[0330:ACPSPA]2.0.CO;2.

[3] Nakabachi A, Ueoka R, Oshima K, Teta R, Mangoni A, Gurgui M, Oldham N, van Echten-Deckert G, Okamura K, Yamamoto K, Inoue H, Ohkuma M, Hongoh Y, Miyagishima S, Hattori M, Piel J, Fukatsu T 2013 Defensive bacteriome symbiont with a drastically reduced genome. Curr Biol 23:1478–1484. doi:10.1016/j.cub.2013.06.027. 23850282

[4] Camacho C, Coulouris G, Avagyan V, Ma N, Papadopoulos J, Bealer K, Madden TL 2009 BLAST+: architecture and applications. BMC Bioinformatics 10:421. doi:10.1186/1471-2105-10-421. 20003500 PMC2803857

[5] Aziz RK, Bartels D, Best AA, DeJongh M, Disz T, Edwards RA, Formsma K, Gerdes S, Glass EM, Kubal M, Meyer F, Olsen GJ, Olson R, Osterman AL, Overbeek RA, McNeil LK, Paarmann D, Paczian T, Parrello B, Pusch GD, Reich C, Stevens R, Vassieva O, Vonstein V, Wilke A, Zagnitko O 2008 The RAST server: Rapid Annotations using Subsystems Technology. BMC Genomics 9:75. doi:10.1186/1471-2164-9-75. 18261238 PMC2265698

